# Control of terahertz nonlinear transmission with electrically gated graphene metadevices

**DOI:** 10.1038/srep42833

**Published:** 2017-02-20

**Authors:** Hyun Joo Choi, In Hyung Baek, Bong Joo Kang, Hyeon-Don Kim, Sang Soon Oh, Joachim M. Hamm, Andreas Pusch, Jagang Park, Kanghee Lee, Jaehyeon Son, Young U. k. Jeong, Ortwin Hess, Fabian Rotermund, Bumki Min

**Affiliations:** 1 Department of Mechanical Engineering, Korea Advanced Institute of Science and Technology (KAIST), Daejeon 305-751, Republic of Korea; 2 Center for Quantum Beam-based Radiation Research, Korea Atomic Energy Research Institute, Daejeon 305-353, Republic of Korea; 3 Department of Physics and Department of Energy Systems Research, Ajou University, Suwon 443-749, Korea; 4 The Blackett Laboratory, Department of Physics, Imperial College, London SW7 2AZ, United Kingdom; 5 Department of Physics, Korea Advanced Institute of Science and Technology (KAIST), Daejeon 305-751, Republic of Korea

## Abstract

Graphene, which is a two-dimensional crystal of carbon atoms arranged in a hexagonal lattice, has attracted a great amount of attention due to its outstanding mechanical, thermal and electronic properties. Moreover, graphene shows an exceptionally strong tunable light-matter interaction that depends on the Fermi level - a function of chemical doping and external gate voltage - and the electromagnetic resonance provided by intentionally engineered structures. In the optical regime, the nonlinearities of graphene originated from the Pauli blocking have already been exploited for mode-locking device applications in ultrafast laser technology, whereas nonlinearities in the terahertz regime, which arise from a reduction in conductivity due to carrier heating, have only recently been confirmed experimentally. Here, we investigated two key factors for controlling nonlinear interactions of graphene with an intense terahertz field. The induced transparencies of graphene can be controlled effectively by engineering meta-atoms and/or changing the number of charge carriers through electrical gating. Additionally, nonlinear phase changes of the transmitted terahertz field can be observed by introducing the resonances of the meta-atoms.

Tunable light-matter interaction is one of most interesting properties of graphene^1^,^2^,^3^. In graphene-based optical devices, the interaction can be controlled by either changing the optical conductivity of graphene or introducing electromagnetic resonances. Whereas the former can be achieved by changing the Fermi level of graphene by chemical^4^,^5^ or electrostatic doping^6^,^7^,^8^,^9^,^10^, the latter involves the techniques of patterning/stacking graphene for manipulating plasmon resonances^7^,^11^ or tuning geometric parameters of metallic meta-atoms that are hybridised with graphene^10^,^12^. These methods are proven to be effective in both the optical terahertz (THz) regimes^7^,^8^,^10^. However, *controllable THz nonlinear* light-matter interaction of graphene has not been observed. Due to the difficulties of generating high fluence THz pulses, the experimental demonstrations of induced transparencies of bare graphene under high THz fluences were reported only recently^13^,^14^,^15^. Theoretical work suggests that the physical mechanism behind the induced transparencies under high THz fluences is the decrease of graphene conductivity due to a reduction of carrier mobility^16^,^17^. Hwang *et al*. supported this prediction by fitting their experimentally measured induced transparency data to calculated results with different momentum scattering times^14^. However, previous investigation is limited in that the controlling mechanisms for THz nonlinearities are not experimentally verified.

Here, we provide experimental proofs of *controllable* nonlinear light-matter interaction of graphene metadevices under illumination of high THz fluences. It is shown that the modulation range of the induced transparencies is strongly influenced by the resonance of metallic meta-atoms patterned on graphene, i.e., whether they are resonant with the exciting light field. Resonant meta-atoms are also shown to introduce nonlinear phase changes of the transmitted light. Moreover, the induced transparencies of graphene metadevices under high THz fluences can be controlled by shifting the Fermi level of graphene, for example, via electrical gating. These schemes of control over nonlinear light-matter interaction in graphene/meta-atom hybrid structures are promising for the realisation of ultra-thin THz devices with controllable nonlinear optical response.

Nonlinear light-matter interaction can be manifested when the light field is strong enough to change the state of the material and its optical response. Therefore, to understand the induced transmittance changes in graphene, we first need to contemplate how the strong light field modifies the carrier distribution and how, in turn, this change affects the transmission of the light field. Above a certain doping level, THz light does not have enough photon energy to induce interband transitions (e.g., 1 THz corresponds to 4.1 meV), so the conductivity is mostly determined by the intraband contribution. The intraband conductivity in the Kubo formula is


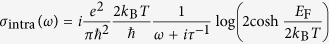


where *τ* is the momentum scattering time, *T* is the carrier temperature, *E*_F_ is the Fermi level of the carrier distribution, and *e*, 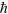, *k*_B_, and *ω* are the elementary charge, the reduced Plank constant, the Boltzmann constant and the angular frequency, respectively^9^. The incident THz photons are partly absorbed by free carriers in graphene by the intraband transitions, and the absorbed energy is quickly redistributed to other carriers in a few tens of femtoseconds by carrier-carrier scattering^18^. By this process, the electron distribution becomes more gradual and is well described by the Fermi-Dirac distribution with electron temperature higher than lattice temperature, the so called “hot-carrier distribution”. In this model, the number of carriers is not substantially changed by the THz field as long as the Fermi-level is higher than half of the energy of the exciting field, but the rate of collision between electrons increases with the temperature of the electron gas. It can be seen from the Kubo formula how the reduction of the momentum scattering time *τ* (or the increase of the collision rate) decreases the real part of conductivity and induces the nonlinear THz response.

The nonlinear response of materials can be amplified by local field enhancements^19^. Metamaterials can be constructed with highly capacitive meta-atoms and strong field enhancement can be induced in their capacitive gaps^20^. The light-matter interaction in the gaps is strengthened due to the local field enhancement, and the material reveals its nonlinear response at low light intensity. The local field enhancement in the metamaterial strongly depends on its geometrical structures and their associated parameters, as does the nonlinear response. Therefore, the enhancement of THz nonlinearity of a material can be achieved by the control of the geometrical parameters of meta-atoms that are hybridised with graphene.

Graphene itself allows for a second way to improve the nonlinear response; namely, a higher carrier concentration leads to a higher conductivity, which in turn allows for stronger nonlinear interaction. According to the hot-carrier generation process explained above, a strong THz field increases the momentum scattering rate of the charge carriers. Conductivity variation due to the change in the momentum scattering rate can be approximately expressed as 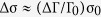, where Γ_0_ and ΔΓ are the initial momentum scattering rate and the change in the momentum scattering rate, respectively^21^,^22^. For the same change of the momentum scattering time, the higher initial conductivity of the graphene produces a larger conductivity change in a proportional manner. The nonlinear THz transmittance change, therefore, can be enhanced by increasing the initial conductivity of graphene or its Fermi level, equivalently.

## Results

**Figure 1:**
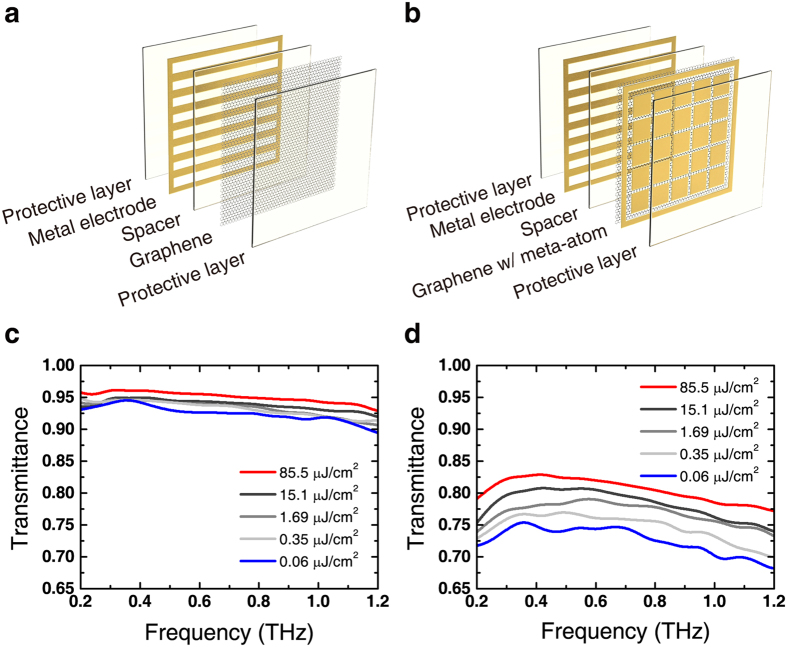
Graphene metadevice structures and THz transmittance spectra. Schematic diagrams of (**a**) a graphene only device (GD) and (**b**) a non-resonant meta-atom/graphene device (NRGD). THz transmittance spectra of (**c**) GD and (**d**) NRGD for different fluences ranging from 0.06 to 85.5 μJ/cm^2^.

To experimentally confirm the nonlinearity controlling mechanisms described above qualitatively, we fabricated electrically gated graphene devices with and without (non-)resonant meta-atoms and measured the response of these devices to high THz fluences. The spectral transmittance was obtained via THz time-domain spectroscopy (THz-TDS) and the power-dependent transmittance was measured by a THz power meter. The structures depicted in Fig. 1a,b comprise a terahertz transparent electrode (TTE), a polyimide spacer, a single graphene layer (or a graphene/meta-atom hybrid layer) that also acts as an electrode, and protective encapsulating polyimide layers on both sides. The Fermi level can be shifted by applying a static electric field between the electrodes. The meta-atoms were designed to show (non-)resonant response within the frequency range of interest (See Fig. 1). High-power THz pulses were generated via optical rectification in a prism-cut LiNbO_3_ crystal capable of generating electric field strengths of ~350 kV/cm. The induced transparency comes from graphene and not from other metallic structure by measurements performed on a sample fabricated without graphene (See Methods and Supplementary Information for a more detailed information on the experimental setup and the design of the devices).

First, we investigated the role of field enhancement in the induced transmittance change of graphene devices by comparing the nonlinear response of a bare graphene device (GD) with a non-resonant meta-atom/graphene device (NRGD). Both devices were fabricated on the same wafer using chemical vapour deposition (CVD) grown graphene that is transferred from the same Cu foil. The THz transmittance data shown in Fig. 1c,d confirm that broadband induced transparency is observed for both devices with increasing THz fluences. For the variation of the incident THz fluence from *F*_THz_ = 0.06 μJ/cm^2^ to *F*_THz_ = 85.5 μJ/cm^2^, the changes in amplitude transmittance at 0.6 THz were measured to be Δ*t*_GD@0.6THz_ = 2.9% and Δ*t*_NRGD@0.6THz_ = 7.8%, respectively. The transmittance dropped because of the increase in reflection and absorption due to the incorporation of metallic meta-atoms, but the change in transmittance was enhanced. It is worth noting that larger nonlinear transmittance change for NRGD is achieved without substantial change in the spectral response.

To confirm that the field enhancement effect leads to increased nonlinearity, we first calculated the average field enhancement factor in the capacitive gap between the meta-atoms by numerical simulations. The average field enhancement factor is defined here as 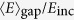, where 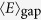 is the averaged electric field in the capacitive gap between the meta-atoms and 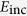 is the electric field of the incident THz wave. For the meta-atom with a lattice constant of 9 μm and a gap width of 1.45 μm, the factor was 3.15. For the maximal change in incident THz fluence, the amplitude transmittance change for the NRGD, Δ*t*_NRGD_, was 2.95 times larger than that for the GD, Δ*t*_GD_. Therefore, our data indicate that the field enhancement by the meta-atoms contributes to the increase in nonlinear transmittance change.

**Figure 2:**
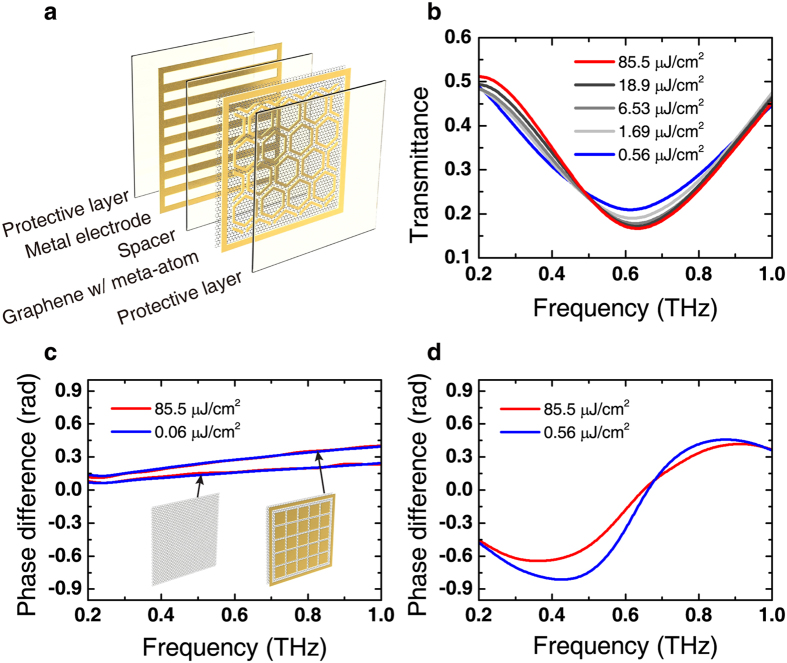
Resonant meta-atom/graphene device and THz transmittance and phase spectra. (**a**) Schematic diagram of resonant meta-atom/graphene devices (RGD). (**b**) THz transmittance spectra of RGD for different fluences ranging from 0.56 to 85.5 μJ/cm^2^. Phase difference spectra through (**c**) GD, NRGD and (**d**) RGD.

In subsequent experiments, we used resonant meta-atom/graphene devices (RGDs) to investigate the additional nonlinear effects, such as the nonlinear phase change or a reversal of the nonlinear transmittance change (i.e., induced opacity) near the resonance frequency. RGD samples were fabricated to have a resonant response at 0.6 THz, as shown in Fig. 2b. Fluence-dependent measurements of the RGD show several distinctive features compared to the results for the NRGD. The most distinguishable feature is the decrease in transmittance near the resonance frequency with increasing incident THz fluence. Such resonant transmittance decrease is accompanied by an off-resonant transmittance increase, and a resonance frequency blue-shift that is also observed when the conductivity of graphene, which is embedded inside the capacitive gap of the meta-atoms, decreases^10^,^20^. The electric field transmittance did not show an enhancement of the nonlinear change because the reverse in transmittance change near resonance counterbalances the amount of off-resonance change. The nonlinear modulation of the amplitude transmittance is strongly frequency-dependent in the resonant device, and there are additional effects not observable for NRGDs. The phase difference of transmitted THz waves was large in the RGD measurements compared to non-resonant devices, which can be seen in the Fig. 2c,d. The RGD, for instance, shows a maximum phase modulation of Δ*ϕ* = 0.25 rad near 0.5 THz, as opposed to the negligible phase change of NRGDs. The large nonlinear phase change is a result of the blue-shift of the resonance under higher THz fluences. The phase response of a meta-atom is highly dispersive in the vicinity of its resonance frequency; hence, a large nonlinear phase change can be observed for a slight resonance blue-shift with increasing fluence. However, for the case of NRGDs, their resonance is designed to be positioned far from the frequency of interest. Therefore, the fluence-dependent phase difference is almost negligible in NRGDs.

**Figure 3:**
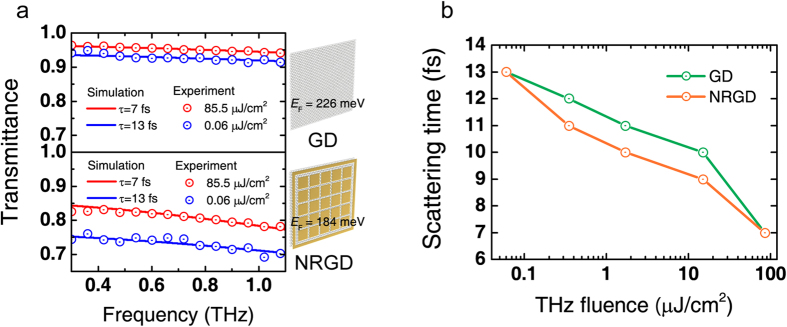
Numerical simulation results of GD and NRGD. (**a**) THz transmittance spectra for GD (top) and NRGD (bottom) from the numerical simulations (solid lines) and the experiments (scatterers). (**b**) Momentum scattering time with respect to the THz fluence calculated by fitting the simulation results to the measurements.

The decrease in momentum scattering time is confirmed by the comparison between the simulations and the experimental results for NRGDs (Fig. 3). The experimentally measured data were fitted to the numerical simulation results so that the transmittance spectra match well throughout the frequency range of interest. For the GD, a good match was achieved with *E*_F_ = −226 meV, whereas the momentum scattering time varied from *τ* = 13 fs at *F*_THz_ = 0.06 μJ/cm^2^ to *τ* = 7 fs at *F*_THz_ = 85.5 μJ/cm^2^, as shown in Fig. 3. From the plot, it can be also confirmed that the momentum scattering was modified with meta-atom hybridisation. In the NRGD fabricated simultaneously with the GD, the range of the fitted momentum scattering time varied from *τ* = 13 fs at *F*_THz_ = 0.06 μJ/cm^2^ to *τ* = 7 fs at *F*_THz_ = 85.5 μJ/cm^2^. Although the scattering parameters are the same with GD for the lowest and the highest fluences, NRGD has lower scattering parameters for other fluences. Note that the Fermi level also altered with 184 meV by the interaction between graphene and gold^23^.

**Figure 4:**
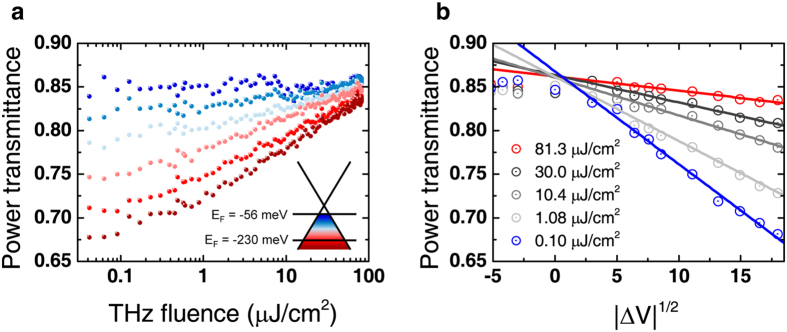
Power transmittance for different Fermi levels of graphene. (**a**) THz power transmittance spectra of GD for different Fermi levels of graphene. The inset indicates the range of variation of the Fermi level. (**b**) THz power transmittance versus square root of the gate voltage (which is proportional to the Fermi level) for different fluences.

Finally, the nonlinear power transmittance change for a GD was investigated as a function of the Fermi level. Without electrical gating, the graphene in the GD was slightly *p*-doped, and the corresponding Fermi level was estimated to be *E*_F_ = −169 meV. Figure 4a shows the Fermi level-dependent power transmittance change as a function of the THz fluence for the GD. For this measurement, the Fermi level was shifted from *E*_F_ = −230 meV to −56 meV. The power transmittance change Δ*T*_EF_ increases as the Femi level is shifted away from the charge neutral point (CNP); for example, the change was Δ*T*_−230 meV_ ~16% and Δ*T*_−56 meV_ <1%. In another plot, Fig. 4b, it is clearly seen that the power transmittances monotonically decrease as the (square root of) gate voltage from CNP with steeper slopes for higher THz fluences. This difference in the power transmittance change can be attributed to the difference in the initial conductivity of free carriers in graphene. For a low doping level, however, the gate voltage dependency disappeared because the momentum scattering rate is not determined by the free carriers but mainly by the impurity concentration, which barely changes with the Fermi level near CNP^10^,^24^. Moreover, the momentum scattering time associated with electron-impurity can be expressed as 
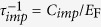
, where 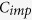 is a constant, and it has no dependency on the electron temperature^25^. In the vicinity of the CNP, therefore, the transmittance stays fairly constant with the THz fluence, which changes the electron temperature, and/or the gate voltage, which changes the free carrier concentration.

## Discussion

We have explored different mechanisms to control the nonlinear transmittance change of graphene metadevices at THz frequencies. Metamaterials concentrates the incident electric field in their gaps between meta-atoms, and the enhanced field can be exploited to control the nonlinear response of graphene. The metadevices can be designed to show either broadband induced transparency or resonant induced opacity by controlling the geometry of the meta-atoms. In addition, dynamic control over the nonlinear response has been demonstrated with a gated graphene sheet. The free carrier concentration of graphene can be controlled by the gate voltage, and a higher free carrier concentration produces a higher nonlinear transmittance change. This work constitutes a crucial early step towards the employment of ultra-thin and controllable graphene devices in nonlinear THz technology.

## Methods

### Fabrication of nonlinear graphene metadevices

The graphene metadevices were fabricated on a sacrificial silicon wafer substrate. Polyimide solution (PI-2610, HD MicroSystems^TM^) was used for the fabrication of protective dielectric layers and a spacer. The PI solution was spin-coated on a silicon wafer to have a final thickness of 1 μm and was then baked. The baking process consisted of two steps: first, the solvent was volatilised in a convection oven, and second, PI was cured in a furnace. All of the metal parts were made of 100-nm-thick gold with a 10-nm-thick adhesive chromium layer. The bottom terahertz transparent electrode was patterned by photolithography and electron beam evaporation deposition followed by metal lift-off. The PI layer as a spacer was stacked using the same procedure described above. Then, the array of meta-atoms was patterned on top of the PI spacer. Single-layer graphene (GRAPHENE SQUARE Inc.) was grown on a copper foil by chemical vapour deposition and was transferred to cover the whole metamaterial area by the thermal release tape method. The top (ground) electrode was fabricated on the graphene layer using a shadow mask. All layers were capped with another PI layer and were delaminated from the silicon wafer.

### Measurements under high THz fluence

To obtain the spectral response in the THz regime, THz time-domain spectroscopy (THz-TDS) was employed. High-power THz pulses were generated with a lithium niobate (LiNbO_3_) crystal with a 1-kHz Ti:sapphire regenerative amplifier (Spitfire Ace, Spectra-physics). The system was capable of generating near-infrared (NIR) pulses with an average power of 4 Watts. The pulse duration was approximately 100 fs, and the centre wavelength was 800 nm. The maximum power of the generated THz pulses and the peak-to-peak electric field amplitude that can be generated by the setup were 3.3 mW and 350 kV/cm, respectively. The spectral response of the graphene metadevices was recorded by the THz-TDS. The time-domain signal of the transmitted THz pulses was obtained by electro-optic sampling with gallium phosphide (GaP) crystal. The total power transmittance was measured by a calibrated pyroelectric detector (PyroCAM-III, OPHIR), and the corresponding amplitude transmittance was calculated by taking the square root of the power transmittance. The fluence of the incident THz wave was varied by changing the relative angle between two THz wire-grid polarisers. The THz fluence could be varied from 0.008 μJ/cm^2^ to 85.5 μJ/cm^2^.

## Additional Information

**How to cite this article:** Choi, H. J. *et al*. Control of terahertz nonlinear transmission with electrically gated graphene metadevices. *Sci. Rep.* **7**, 42833; doi: 10.1038/srep42833 (2017).

**Publisher's note:** Springer Nature remains neutral with regard to jurisdictional claims in published maps and institutional affiliations.

## Supplementary Material

Supplementary Information
