# Supplementary material for: Control of terahertz nonlinear transmission with electrically gated graphene metadevices
Source: Sci Rep. 2017 Feb 20;7:42833. doi: 10.1038/srep42833 (PMC5316969; doi:10.1038/srep42833)
Supplement: Supplementary Information [file srep42833-s1.pdf]

SUPPLEMENTARY INFORMATION

for

"Control of terahertz nonlinear transmission with electrically gated graphene metadevices"

Hyun Joo Choi[1,3*], In Hyung Baek[2,3*], Bong Joo Kang[2], Hyeon-Don Kim[1], Sang Soon Oh[4], Joachim M. Hamm[4], Andreas Pusch[4], Jagang Park[1], Kanghee Lee[1], Jaehyeon Son[1], Young Uk Jeong[3], Ortwin Hess[4], Fabian Rotermund[2,5#] & Bumki Min[1#]

[1]*Department of Mechanical Engineering, Korea Advanced Institute of Science and Technology (KAIST), Daejeon 305-751, Republic of Korea*

[2]*Department of Physics and Department of Energy Systems Research, Ajou University, Suwon 443-749, Korea*

[3]*Center for Quantum Beam-based Radiation Research, Korea Atomic Energy Research Institute, Daejeon 305-353, Republic of Korea*

[4]*The Blackett Laboratory, Department of Physics, Imperial College, London SW7 2AZ, United Kingdom*

[5]*Department of Physics, Korea Advanced Institute of Science and Technology (KAIST), Daejeon 305-751, Republic of Korea*

[*]These authors contribute equally to this work.

[#]Corresponding authors: rotermund@kaist.ac.kr, bmin@kaist.ac.kr

1. Characterization of graphene

In order to characterize the transferred CVD-grown graphene, Raman spectroscopy was used with a 514 nm excitation laser. The representative features of the SLG are (1) the 2D-peak over G-peak intensity ratio (I_{2D}/I_G) is generally in the range between 2 and 4, and (2) the full width half maximum (FWHM) of 2D-peak is about ~30 cm^{-1}. As shown in Fig. S1a, our graphene sample has I_{2D}/I_G ratio of 3.3 and FWHM of 30.2 cm^{-1} at the Raman shift of 2685.8 cm^{-1}. It satisfied above features of SLG. To further confirm the uniformity of graphene, we also conducted Raman spectroscopic mapping of I_{2D}/I_G for CVD-grown graphene over an arbitrarily chosen area of 20 × 20 μm^2, and the data are presented in Fig. S1b.

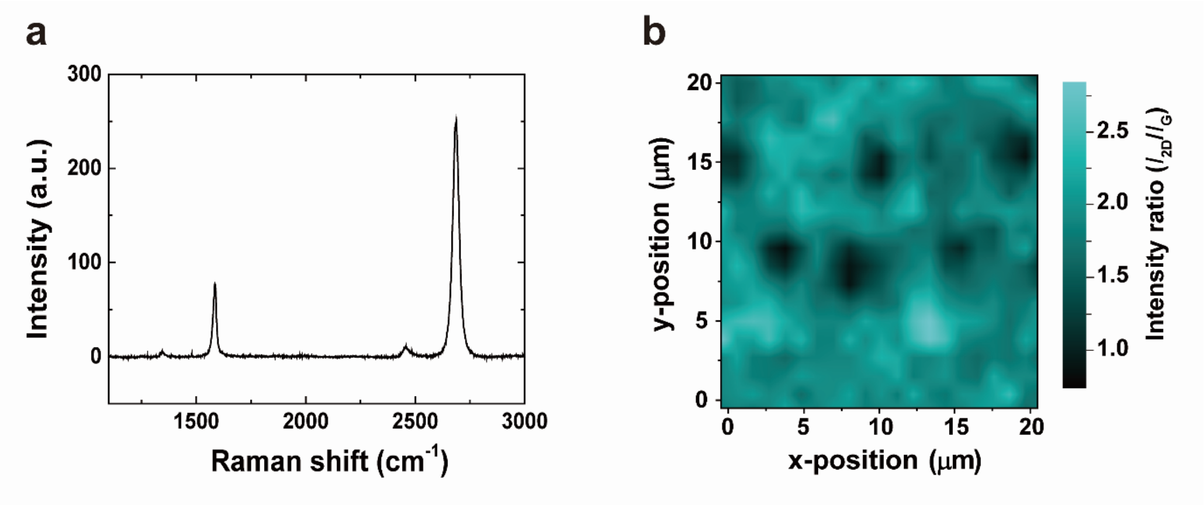

Figure S1. **(**a) Raman intensity plot of CVD-grown graphene shows that 2D peak is located at 2685.8 cm^{-1} with the FWHM of about 30.3 cm^{-1}. (b) Mapping of Raman intensity ratio of G-peak to 2D-peak (I_{2D}/I_G) for arbitrarily chosen area of 20 × 20 μm^2 of CVD-grown graphene.
